# PSMA-Specific CAR-Engineered T Cells Eradicate Disseminated Prostate Cancer in Preclinical Models

**DOI:** 10.1371/journal.pone.0109427

**Published:** 2014-10-03

**Authors:** Gaia Zuccolotto, Giulio Fracasso, Anna Merlo, Isabella Monia Montagner, Maria Rondina, Sara Bobisse, Mariangela Figini, Sara Cingarlini, Marco Colombatti, Paola Zanovello, Antonio Rosato

**Affiliations:** 1 Department of Medicine, University of Padua, Padua, Italy; 2 Department of Pathology and Diagnostics, Section of Immunology, University of Verona, Verona, Italy; 3 Veneto Institute of Oncology IOV - IRCCS, Padua, Italy; 4 Department of Surgery, Oncology and Gastroenterology, University of Padua, Padua, Italy; 5 Ludwig Center for Cancer Research, University of Lausanne, Lausanne, Switzerland; 6 Molecular Therapies Unit, Department of Experimental Oncology and Molecular Medicine, Fondazione IRCCS Istituto Nazionale dei Tumori, Milan, Italy; 7 Medical Oncology, Azienda Ospedaliera Universitaria Integrata (AOUI), Verona, Italy; Baylor College of Medicine, United States of America

## Abstract

Immunology-based interventions have been proposed as a promising curative chance to effectively attack postoperative minimal residual disease and distant metastatic localizations of prostate tumors. We developed a chimeric antigen receptor (CAR) construct targeting the human prostate-specific membrane antigen (hPSMA), based on a novel and high affinity specific mAb. As a transfer method, we employed last-generation lentiviral vectors (LV) carrying a synthetic bidirectional promoter capable of robust and coordinated expression of the CAR molecule, and a bioluminescent reporter gene to allow the tracking of transgenic T cells after *in vivo* adoptive transfer. Overall, we demonstrated that CAR-expressing LV efficiently transduced short-term activated PBMC, which in turn were readily stimulated to produce cytokines and to exert a relevant cytotoxic activity by engagement with PSMA^+^ prostate tumor cells. Upon *in vivo* transfer in tumor-bearing mice, CAR-transduced T cells were capable to completely eradicate a disseminated neoplasia in the majority of treated animals, thus supporting the translation of such approach in the clinical setting.

## Introduction

Adoptive cell therapy with unmodified and *ex vivo* expanded T cells has proved effective against different tumor entities, in particular melanoma and EBV-associated tumors [1]. Nowadays, the genetic modification of T cells can confer new tumor-targeting properties to naturally occurring lymphocytes, thus overcoming the reliance on components of the endogenous immune system.

While transduction with Ag-specific TCR can only redirect T cell activity based on the same recognition characteristics, Chimeric Antigen Receptor (CAR) technology has the potentiality to endow T cells with the advantageous features of an antibody, namely specificity, affinity and the possibility to target non-protein antigens [2]. Moreover, in an unique molecule CAR provides some measures to counteract tumor immune evasion strategies: it relieves T cell recognition and activity of MHC restriction and expression, and can relay costimulatory signals through its intracellular domains.

Therapeutic efficacy of CAR T cells have been already reported in patients, in particular against chronic lymphocytic leukemia (CLL) and acute lymphoblastic leukemia (ALL) [3]–[6] with very promising results in terms of disease free survival and complete hematological and molecular responses even in subjects who failed all previous standard treatments. However, hematological malignancies, in particular those of B cell origin, can be regarded as an ideal target for immunotherapeutic approaches [7]. Indeed, these malignant cells naturally provide costimulatory receptor ligands and share the same physiological compartments with adoptively transferred T cells. Finally, elimination of normal B cells is associated with non life-threatening adverse effects, which can be clinically managed with intravenous immunoglobulin administration.

Conversely, solid tumor treatment remains a major challenge and should be improved both in terms of clinical efficacy and safety. Partial successes were experienced against neuroblastoma using GD2-specific CAR T cells without pre-conditioning regimen, in the virtual absence of side effects [8], [9]. By contrast, no clinical responses were recorded with the infusion of T cells redirected against the folate receptor in ovarian carcinoma patients [10], nor against carboxy-anhydrase IX (CAIX) in renal cell carcinoma patients [11], despite the relevant “on-target, off-tumor” toxicity evidenced in the latter case. Lessons learned from these experiences indicate that the definition of the target antigen for safety issues, and the persistence and tumor homing capacity of infused cells are particularly critical for a successful treatment.

We addressed some of these questions by targeting prostate tumor cancer cells with T cells modified to express a CAR specific for the human prostate-specific membrane antigen (hPSMA).

Prostate tumor represents a serious clinical entity, with estimated 233,000 new cases and 29,480 deaths in U.S. in 2014 [12], with at present only palliative treatments for hormone refractory and metastatic forms [13]. For these patients, immunotherapy has proved to be a valid option based on vaccination with modified whole prostatic tumor cells (GVAX [14]) or PBMC presenting a relevant prostatic antigen (Sipuleucel-T [15]). With regard to adoptive cell therapy approaches, preclinical studies have reported encouraging results [13], [16], [17] and clinical evaluation is undergoing (clinical trials number NCT01140373, NCT01929239, NCT00664196; www.clinicaltrials.gov). In this scenario, PSMA can represent a suitable target and indeed it is currently exploited for both imaging and therapeutic purposes. In particular, PSMA expression levels differentiate normal and cancerous prostatic tissues, and parallel the Gleason score of prostate cancer [18]. Interestingly, PSMA expression involves neovasculature of several tumor entities, thus envisaging an additional antiangiogenic effect.

Here, we report the design of CAR against hPSMA based on a novel and high affinity specific mAb [19], and the phenotypic and functional characterization of T-body-hPSMA both *in vitro* and *in vivo*. For transduction, we used a lentiviral vector carrying a bidirectional promoter which drives the simultaneous expression of the CAR molecule and a reporter bioluminescent gene. This allowed the tracking of infused T cells and hence the direct correlation of the therapeutic outcome with the persistence and homing capacity of T cells. Overall, CAR-transduced T cells not only exerted a relevant loco-regional therapeutic activity, but also completely eradicated a disseminated neoplasia in the majority of treated animals. These results strongly support the translation of such approach in the clinical setting.

## Materials and Methods

### Cell lines

The following cell lines were used in this study: LNCaP and PC3, human prostate carcinoma cell lines; Jurkat, human T lymphoblastic leukemia and 293 T [20], human embryonic kidney cell line. The PC3-PIP cell line, stably expressing human PSMA (hPSMA) [21] was generously provided by Dr. Warren Heston; LNCaP cell line was obtained from American Type Culture Collection (ATCC, Rockville, MD, USA). LNCaP, PC3, PC3-PIP and Jurkat cells were maintained in RPMI 1640 medium (EuroClone, Milan, Italy), while DMEM medium (Biochrom AG, Berlin, Germany) was used for 293 T cells; both media were supplemented with 10% Fetal Bovine Serum (FBS, Gibco BRL Paisley, UK), 2 mM L-Glutamine, 10 mM HEPES, 100 U/ml Penicillin, and 100 U/ml Streptomycin (all from Lonza, BioWhittaker, Basel, Switzerland), at 37°C in a 5% CO_2_ atmosphere.

### Anti-hPSMA CAR generation

The CAR against the hPSMA antigen contains the complete sequence of the anti-hPSMA scFv derived from the anti-hPSMA D2B hybridoma [19]. This sequence was cloned into the multiple cloning site (MCS) of the pSecTag2A vector (Invitrogen, San Giuliano Milanese, Milan, Italy). The MCS of the pSecTag2A plasmid is comprised between two sequences: in 5′, the murine Ig kappa light chain leader sequence V-J2-C, and in 3′ the Myc Tag sequence. Thus, the leader-ScFv-myc sequence was used for CAR generation. The Leader-ScFv-myc fragment was amplified by PCR and inserted in the pBS SKII construct (Invitrogen). A portion of the CD28 (bases 438–759) and the CD3ζ intracellular domain (227–563 region), both obtained from cDNA of EBV-specific CD4^+^ T cells [22], were fused by PCR and then inserted into the pBS SKII vector, downstream the Leader-ScFv-myc fragment. This vector was then sequenced at the Centro Ricerca Interdipartimentale Biotecnologie Innovative (CRIBI) of Padua University, Italy. The anti-hPSMA CAR sequence was finally subcloned into the pcDNA3.1 vector (Invitrogen). To check the capacity to drive the expression of the CAR molecule on the membrane, this vector was used to transfect 293 T cells by using Lipofectamine 2000 (Invitrogen), according to the manufacturer’s instructions.

### LV plasmids and lentiviral preparation

The following lentiviral packaging vectors were used: pMDLg/pRRE, pRSV-Rev, pMD2.VSVG and pADVantage, all kindly provided by Dr. L. Naldini (San Raffaele, Milan, Italy). The transfer vector #945.pCCL.sin.cPPT.SV40ployA.eGFP.minCMV.hPGK.deltaLNGFR.Wpre is a self-inactivating (SIN) HIV-derived vector [23], which carries a minCMVPGK divergent bidirectional promoter driving the simultaneous expression of two genes in antisense orientation. The transfer vectors used in this study carried the anti-hPSMA CAR sequence (obtained from pMA-T-body vector) under the control of the hPGK promoter, and the eGFP (enhanced Green Fluorescent Protein) or Firefly Luciferase (fluc) reporter genes under the control of minCMV. pMA-T-body vector and fluc sequence were synthetized by GeneArt, Life Technologies (Regensburg, Germany). Lentiviral production in 293 T cells has been previously described [23]. A lentiviral vector coding only for fluc [24] was used to transduce PC3-PIP cells.

### Western blot analysis

293 T cells, untransfected or transfected with pcDNA3.1-CAR, were lysed in buffer containing 50 µM Tris HCl pH 6.8, 2% sodium dodecyl sulfate (SDS), 2% β-Mercaptoethanol, 10% glycerol, and 0.1–0.05% Bromophenol Blue (al from Sigma-Aldrich, St. Louis, MO, USA) Proteins were separated on 10% SDS-PAGE gels and transferred to PVDF membrane (Immobilion-P, Millipore, Billerica, MA, USA). The membrane was blocked in 5% skimmed milk (Sigma-Aldrich) in TBS and 0.05% Tween 20, followed by incubation with the primary antibody (mouse anti-c-Myc mAb, 1∶1000, Sigma-Aldrich) for one hour at room temperature. After three 10-minutes washes in PBS-Tween, HRP-conjugated goat anti-mouse IgG secondary antibody (diluted 1∶10000, Amersham, Milan, Italy) was added for one hour at room temperature in milk. The membrane was developed using the SuperSignal West Pico (Pierce, Rockford, IL, USA) and visualized using chemiluminescence. Signal intensity was measured using a Bio-Rad XRS chemiluminescence detection system (Life Science Group, Milan, Italy).

### Jurkat cell transduction

To assess the functionality of LV vectors, Jurkat cells were incubated with viral supernatant (hPSMA/eGFP LV CAR or hPSMA/Luciferase LV CAR) for 15 hours in the presence of 8 µg/ml protamine sulfate (Sigma-Aldrich). Flow cytometry and Bioluminescence (BLI) analyses were carried out at different time points post transduction to evaluate CAR and eGFP expression or luciferase activity.

### T-bodies generation

To generate T-bodies, PBMC from healthy donors were activated 48 hours with OKT-3 (50 ng/ml; Ortho Biotech Inc, Raritan, NJ, USA) and human IL-2 (hIL-2, 300 U/ml; Proleukin; Novartis Pharmaceuticals, Horsham, UK). T cells were then infected with the viral supernatant for 18 hours at 37°C and 5% CO_2_, in the presence of protamine sulfate (40 µg/ml; Sigma-Aldrich) and hIL-2 (500 U/ml). The supernatant was then changed with fresh complete medium containing hIL-2 (100 U/ml). Seventy-two hours later, PBMC were analyzed for CAR and eGFP expression, and for luciferase activity. PBMC were referred to as T-body-hPSMA/eGFP and T-body-hPSMA/fluc when transduced with hPSMA/eGFP LV CAR or hPSMA/Luciferase LV CAR, respectively. T-bodies were re-stimulated once a week with irradiated (60 Gy) PC3-PIP at a 10∶1 ratio. Complete medium with fresh IL-2 was replenished twice a week.

### Antibodies

CAR-expressing cells were labeled with the anti-c-myc mAb (clone 9E10; Sigma-Aldrich) or the isotype control (mouse IgG1, Southern Biotech, Milan, Italy), followed by a secondary antibody (PE-conjugated goat anti-mouse IgG; Southern Biotech). Cell surface markers were labeled using APC-, FITC- or PE-conjugated antibodies to CD4, CD8, CD57, CD27, CD28, CD62L (BioLegend, San Diego, CA, USA), CCR7 (eBioscience, San Diego, CA, USA), and the relative isotype controls purchased from the same companies.

### Cytotoxicity assay

The cytotoxic activity of T-body-hPSMA/eGFP and T-body-hPSMA/fluc was assessed in a standard 4 h ^51^Cr-release assay as previously reported [25], at different time points post-transduction. PC3, PC3-PIP and LNCaP were used as target cells.

### Cytokine release assays

To evaluate IFN-γ production, an ELISA IFN-γ Screening Set (Thermo Scientific, Rockford, IL, USA) was used, according to manufacturer’s instructions. Briefly, 1×10^6^ T cells were seeded with 1×10^6^ target cells (PC3 or PC3-PIP) in triplicate wells in 96-well round bottom plates. Cytokine secretion was measured after 12 hours of incubation using T-bodies at different stages of differentiation. Negative and positive controls were represented by untransduced PBMC and T-bodies unstimulated or treated with 40 ng/ml of PMA and 4 µg/ml of Ionomycin (Sigma-Aldrich), respectively. Supernatants were then analysed on a VICTOR X4 (PerkinElmer, Zaventem, Belgium).

### Mice and *in vivo* experiments

*In vivo* experiments involved 6 to 8 week-old males SCID, Rag2^−/−^/γc^−/−^ and NOD/SCID mice (Charles River Laboratories, Calco, Como, Italy), which were housed in the specific pathogen-free animal facility of the Department of Surgery, Oncology and Gastroenterology, Padua University (Italy). Animals were housed with a 12- hour light/dark cycle, in temperature (22+/−1°C) and humidity (55+/−5%) controlled room. All mice were allowed free access to water and a maintenance diet. All cages housed up to 6 animals and contained wood shavings and a cardboard tube for environmental enrichment. Procedures involving animals and their care were in conformity with institutional guidelines that comply with national and international laws and policies (D.L. 116/92 and subsequent implementing circulars), and the experimental protocol (n°7/2012) was approved by the local Ethical Committee of Padua University (CEASA). During *in vivo* experiments, animals in all experimental groups were examined daily for a decrease in physical activity and other signs of disease; severely ill animals (weight loss exceeding 15%, lethargy, ruffled hair, low temperature) were euthanized by carbon dioxide overdose.

#### Winn assay

Winn assay was performed by injecting s.c. SCID mice with 5×10^6^ PC3 or PC3-PIP tumor cells per animal, mixed with either RPMI or T-body-hPSMA/eGFP cells (5×10^6^/mouse; 6 mice/group). Tumor volume was calculated according to the following equation: V (mm^3^) = (d^2^ * D)/2, where d (mm) and D (mm) are the smallest and largest perpendicular tumor diameters, respectively, as assessed by caliper measurement.

#### Loco-regional treatment

To evaluate the therapeutic potential of the loco-regional treatment, SCID mice were injected s.c. with 5×10^6^ PC3-PIP cells. When tumors become palpable about four days later, mice were randomly assigned to the control group (they were left untreated) or the experimental group (they were injected intralesionally with CAR-transduced T cells at 72 hours after transduction; 6 mice/group). Two primary outcomes were analyzed: tumor growth was monitored over time by caliper measurement and the overall survival was recorded.

#### Systemic treatment of subcutaneous prostate tumors

To assess the therapeutic activity of the systemic T-body administration, SCID mice were injected s.c. with 5×10^6^ PC3-PIP cells and 4 days later they received i.v. T-body-hPSMA/fluc (10×10^6^/mouse) at 72 hours or 5–6 weeks post transduction (n = 6); untreated animals served as control group (n = 6). T cell biodistribution was assessed by bioluminescence imaging (BLI) in the same conditions, except that mice were injected with both PC3-PIP or PC3 tumor cells on the right and left flank, respectively.

#### Disseminated prostatic tumor model

To set up a model of disseminated prostatic tumor, SCID, Rag2^−/−^/γc^−/−^ and NOD/SCID mice were injected i.v. with different numbers (ranging from 1×10^5^ to 5×10^6^) of fluc-transduced PC3-PIP cells (6 mice/group). Tumor engraftment and growth were evaluated by BLI. Moreover, tumor-free SCID, Rag2^−/−^/γc^−/−^ and NOD/SCID mice were injected with 20×10^6^ T-body-hPSMA/fluc, to evaluate their fate by BLI (6 mice/group).

#### Adoptive immunotherapy experiments

In adoptive immunotherapy experiments, bioluminescent PC3-PIP were injected i.v. in NOD/SCID and Rag2^−/−^/γc^−/−^ mice (1×10^5^/mouse). Four days later, animals were randomly assigned to the control group (they were left untreated) or the experimental group, where they received i.v. 20×10^6^ T-body-hPSMA/eGFP for 3 times within a week (6 mice/group). Tumor growth was monitored weekly by BLI, and survival was recorded.

### Quantitative Bioluminescence

Quantitative BLI is a powerful technology that allows to obtain multiple images longitudinally and, at the same time, to reduce animal numbers. Bioluminescence images were collected with the IVIS Lumina II Imaging System (PerkinElmer). Ten to 15 minutes before imaging, animals were anesthetized with isoflurane/oxygen and administered i.p. with 150 mg/kg of D-luciferin (PerkinElmer) in PBS. Three mice were imaged simultaneously with exposure times ranging from 0.5 to 3 min. A 12×12 cm field of view and low, medium, or high binning levels were applied to maximize sensitivity and spatial resolution. Ventral images were obtained for each animal and quantified through the region of interest (ROI). Living Image Software (PerkinElmer) was used to acquire and quantify the bioluminescence imaging data sets.

### Cytofluorimetric evaluation of hPSMA expression in growing tumors

Tumor cells suspensions were obtained by enzymatic digestion with 270 units/ml DNase, 35 U/ml hyaluronidase, and 200 U/ml collagenase buffer (all from Sigma-Aldrich). After a 30-min incubation at 37°C, the cells were passed through a 70-µm cell strainer (BD Biosciences, San Jose, CA, USA). Samples were then stained at 4°C with a mouse anti-hPSMA mAb [19] followed by a PE-conjugated anti-mouse IgG1 secondary antibody (BioLegend) and analyzed on a FACSCalibur (BD) flow cytometer. Data were evaluated with FlowJo software (TreeStar Inc., Olten, Switzerland).

### Statistical analysis

Kaplan–Meier product-limit method was performed to estimate the survival curves, and comparison of survival between groups was performed using the log-rank test. Statistical analyses were carried out with the Sigmaplot (version 12.3) statistical package.

## Results

### Construction and development of an efficient bidirectional LV for anti-hPSMA CAR expression

**Figure 1:**
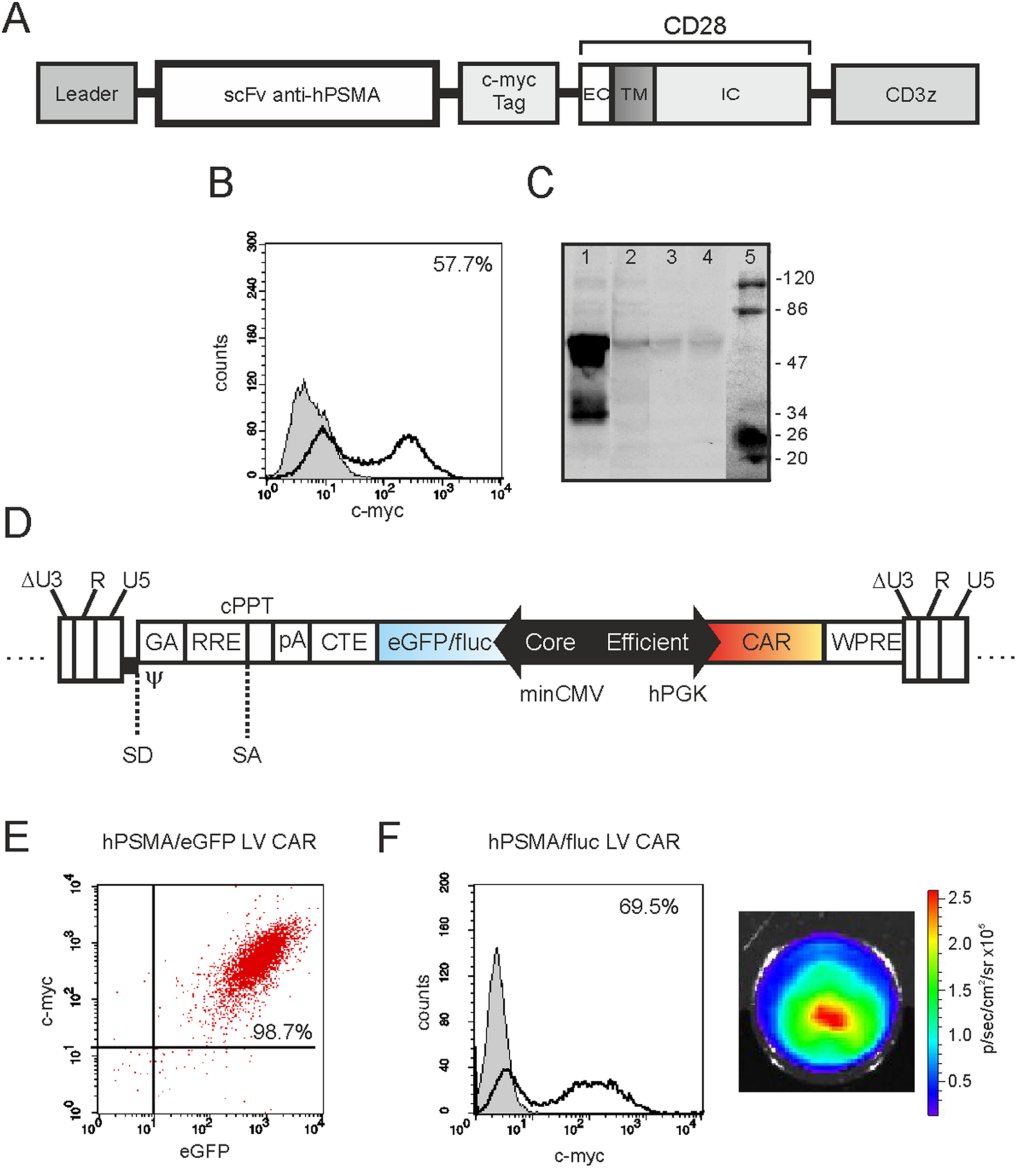
CAR development and validation. (A) Anti-hPSMA CAR map. EC: extracellular domain; TM: transmembrane domain; IC: intracellular domain. (B) Cytofluorimetric profile of CAR expression in transfected 293 T cells. 293 T cells were transfected with pcDNA3.1 vector bearing CAR sequence and analysed by flow cytometry after 24 hours (dark line). Untransfected cells (grey plot) served as control. (C) CAR expression as assessed by Western blotting at 24 hours (line 1) and 7 days (line 2) post 293 T cell transfection. Negative controls were the medium alone (line 3) and untransfected 293 T cells (line 4). Line 5, molecular weight markers. (D) Linear map of the recombinant viral vector containing the minCMVPGK bidirectional promoter (22). In particular, the reporter gene (eGFP or Luciferase) is under the control of minCMV promoter, while the CAR gene is under control of hPGK promoter. (E) Transduction of Jurkat cells with LV CAR anti-hPSMA/eGFP. Co-expression of c-myc and eGFP in LV-transduced Jurkat cells, as assessed by flow cytometry. Dot plot reports the events gated on total viable cells; more than 90% of cells co-express both c-myc and eGFP. (F) Transduction of Jurkat cells with LV CAR anti-hPSMA/Luciferase. *Left panel*, c-myc expression (black) in Jurkat cells at 72 h post transduction, as assessed by flow cytometry. Grey plot represents the isotype control. *Right panel*, Assessment of luciferase activity in LV-transduced Jurkat cells (2×10^5^/well) by BLI.

A CAR sequence was designed that encoded the following components in-frame from 5′ to the 3′ ends (Fig. 1A): a leader sequence, the single-chain variable fragment (ScFv) of the new anti-hPSMA antibody IgGD2B [19], the c-myc tag sequence, and the CD28 costimulatory molecule linked to the CD3ζ sequence. We decided to exploit this new anti-PSMA scFv (scFvD2B) for the construction of our CAR, because of its very appealing characteristics, in particular the nanomolar affinity for the target that is similar to the one evidenced by the whole J591 antibody [19]. Moreover, our scFvD2B shows high specificity and identifies a different epitope from that recognized by the J591 antibody, as assessed by competition experiments carried out with radio-immunoligands or biotin-labeled antibodies ([19] and data not shown).

To assess the capacity of the construct to drive the expression of a receptor that correctly mounted on the cell membrane, the anti-hPSMA CAR sequence was cloned into the pcDNA3.1 vector and the resulting plasmid was used for transient transfection of 293 T cells. At different time points thereafter, the expression of the chimeric receptor was assessed by cytofluorimetric and Western blotting analysis (Fig. 1B and 1C, respectively), using the anti-c-myc mAb. Results disclosed that the CAR was correctly mounted on the membrane and had the expected molecular weight (about 60 kDa); CAR expression was already detectable 24 hours post-transfection, to rapidly disappear the next days due to the lack of a selection step (Fig. 1C). Subsequently, the CAR sequence was inserted into a LV carrying a bidirectional promoter that allowed transcription of a reporter gene (eGFP or fluc) from the upstream minimal promoter minCMV, without affecting downstream expression of the anti-hPSMA CAR from the efficient hPGK promoter (Fig. 1D). To assess functionality of the lentiviral vectors, Jurkat cells were transduced with LV CAR hPSMA/eGFP and LV CAR hPSMA/fluc. CAR turned out to be highly expressed already 72 hours post-transduction, as demonstrated by the high percentage of c-myc^+^ cells detected by flow cytometry analysis (Fig. 1E and 1F). In particular, CAR-expressing cells were more than 90% when transduced with LV CAR hPSMA/eGFP (Fig. 1E) and more than 60% when using LV CAR hPSMA/fluc (Fig. 1F, *left panel*). Notably, the bidirectional LV vectors appeared to drive the expression of either the CAR and the reporter genes with a very balanced efficiency, as demonstrated by the high intensity of eGFP signal (Fig. 1E) and the relevant Luciferase activity (Fig. 1F, *right panel*).

### Phenotypic characterization of CAR-transduced T cell populations

**Figure 2:**
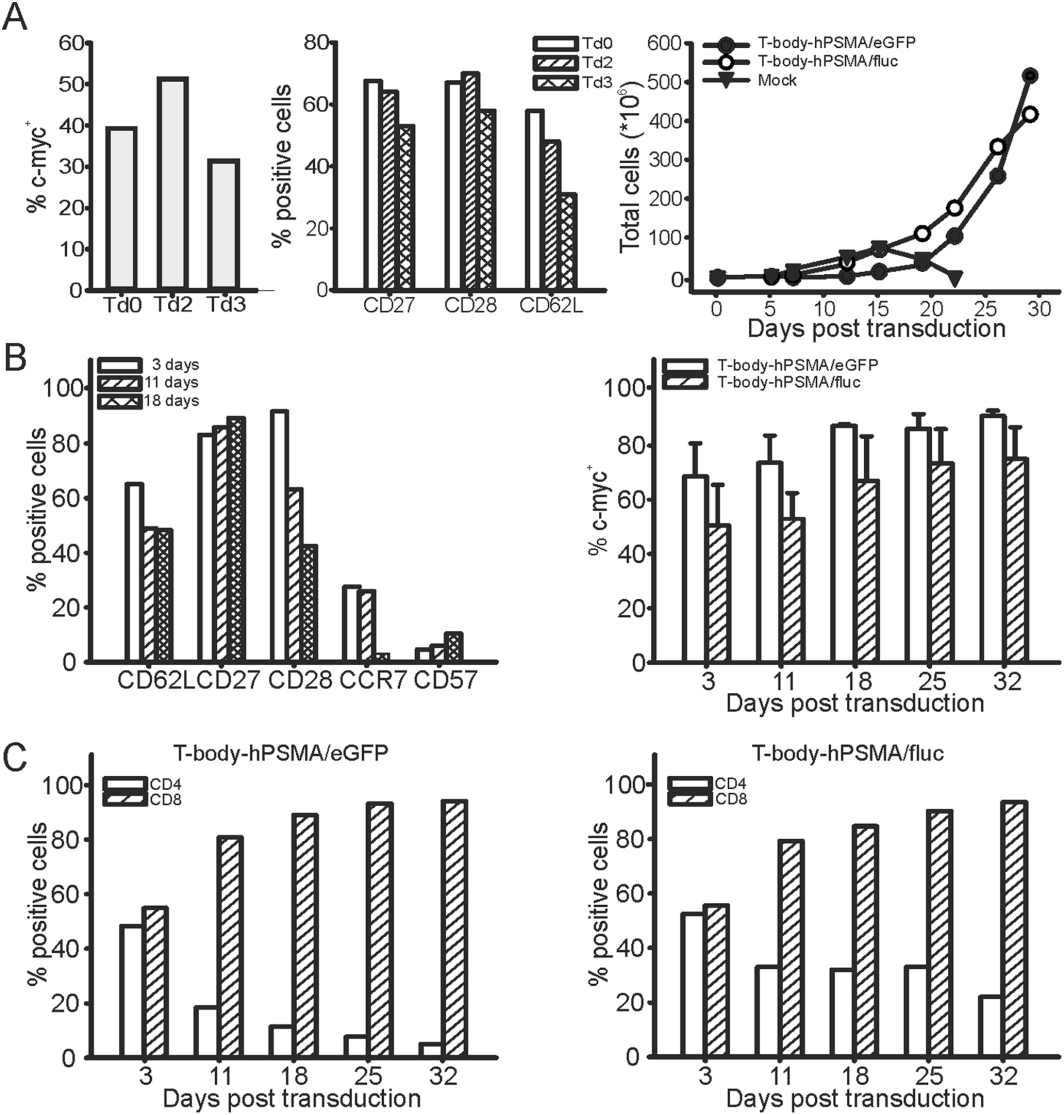
Generation, expansion and phenotipic properties of T-bodies. (A) Set up of transduction protocol and growth kinetics. *Left panel*, PBMC were activated with OKT3 at time 0 and transduced concomitantly (Td0), or after 48-h (Td2) or 72-h (Td3) hours. Flow cytometry analysis of c-myc tag expression was performed 72 hours post infection. *Central panel* reports the expression of CD27, CD28 and CD62L in the three different conditions of activation/infection reported in the *left panel*. *Right panel,* Expansion of T-body-hPSMA/fluc and T-body-hPSMA/eGFP transduced after 48 h activation in response to weekly restimulation with PC3-PIP cells. Untransduced PBMC were used as control. (B) Phenotype and CAR expression in transduced T cells upon stimulation. *Left panel*, Expression of surface markers in T-body-anti-hPSMA/fluc at different time points (3, 11 and 18 days) post transduction, as assessed by flow cytometry. Data are representative of 3 independent experiments. Overlapping results were obtained in T-body-anti-hPSMA/eGFP. *Right panel,* percentage of c-myc^+^ cells in LV CAR hPSMA/fluc and LV CAR hPSMA/eGFP-transduced T cell populations at different time points post transduction. Figure shows mean +/− SD of at least three independent experiments. (C) Kinetics of CD4 and CD8 subsets in transduced T cell populations. Expression of CD4 and CD8 markers in c-myc^+^-gated T-body-hPSMA/eGFP *(left panel)* and T-body-hPSMA/fluc (*right panel)* populations at different time points post transduction, as assessed by flow cytometry. Data are from a representative experiment out of three that produced similar results.

For the generation of T cell populations expressing the anti-hPSMA CAR, a rapid expansion protocol was developed. The optimal infection time was set up after 48 hours of OKT3 activation, since at this time point we obtained the highest percentage of CAR-expressing T cells (Fig. 2A, *left panel*), and a less differentiated phenotype, as assessed by the high expression of CD27, CD28 and CD62L markers (Fig. 2A, *central panel*). Subsequently, the expansion protocol involved weekly restimulation with PC3-PIP cells that allowed a rapid and sustained proliferation of both T-body-hPSMA/eGFP and T-body-hPSMA/fluc populations (Fig. 2A, *right panel*).

To better define the state of differentiation of CAR-transduced T lymphocytes in the post infection period during antigenic restimulations, expression of different surface markers (CD62L, CD27, CD28, CCR7, CD57) was assessed by cytofluorimetric analysis. Seventy-two hours after transduction, the emerging profile was essentially of early effector T cells, as shown by the high expression of CD62L, CD27 and CD28, the presence of CCR7 positive cells and the low expression of CD57 (Fig. 2B, *left panel*). Following restimulations with the antigen, T cells acquired an intermediate effector memory phenotype with the progressive down-modulation of CD62L, CD28 and CCR7 and a slight increase in CD57 expression (Fig. 2B, *left panel*). Interestingly, whereas the subsequent encounter with the antigen led to the expansion of the CAR-expressing population (Fig. 2B, *right panel*), a dichotomy could be observed between the expanding T cell subsets. Indeed, while CAR expression was almost equal within the CD4^+^ and CD8^+^ T cell populations immediately after infection, the antigenic restimulation determined the progressive accumulation of the CD8^+^ T cell subset only, which almost completely overcame CD4^+^ T cells by month one after transduction (Fig. 2C).

### Functional characterization of CAR-expressing T cells

**Figure 3:**
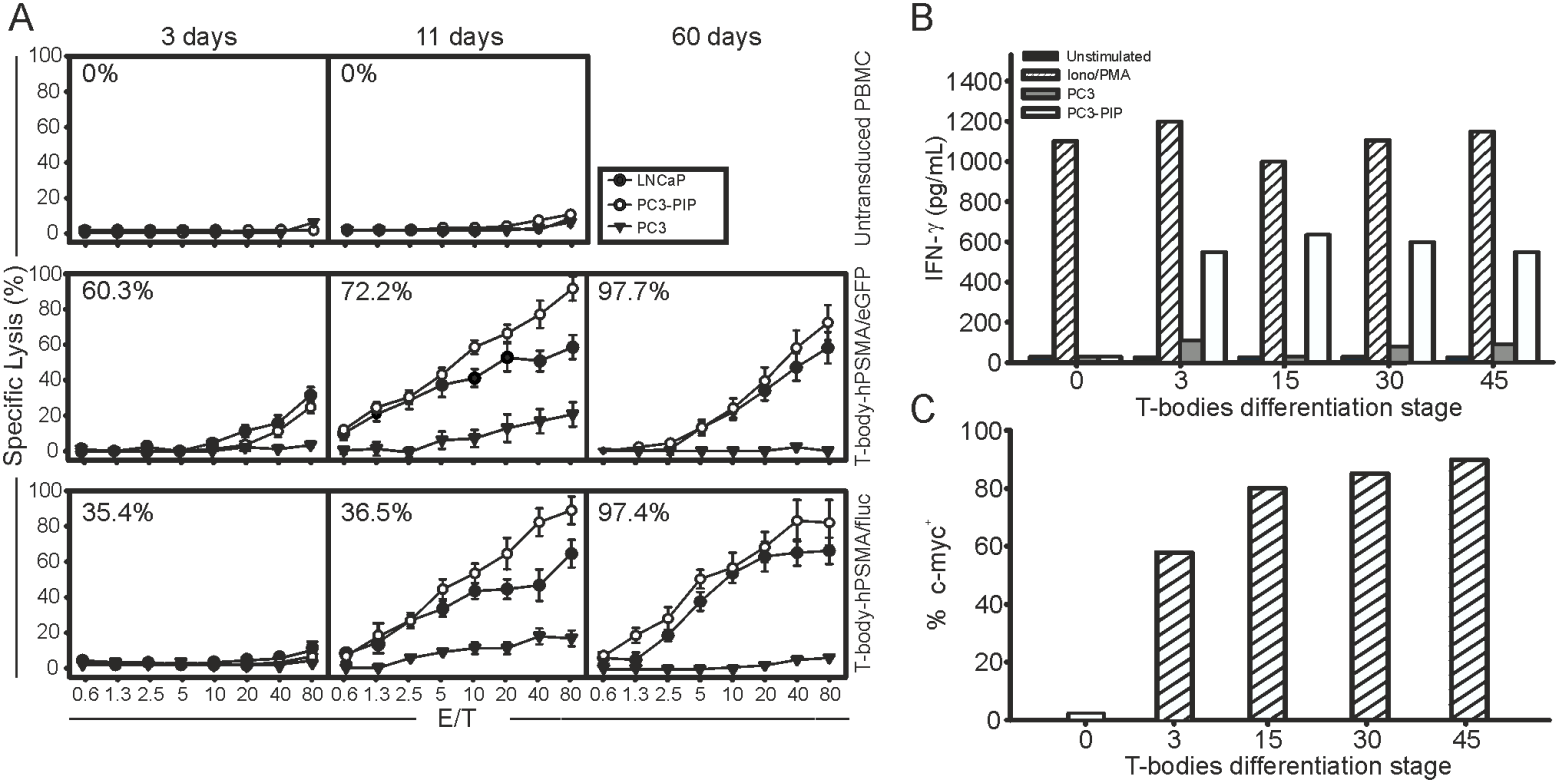
Functional characterization of T-bodies. (A) Lytic activity of T-body-hPSMA/eGFP and T-body-hPSMA/fluc. Cytotoxicity was analysed at 3, 11 and 60 days post-transduction; PC3, PC3-PIP and LNCaP cells were used as target cells. Untransduced PBMC served as negative control. In the upper left corner of each panel the percentages of c-myc^+^ T cells are reported. Figure shows mean +/− SD of 4 independent experiments. (B) IFN-γ secretion upon antigen stimulation. IFN-γ production was analyzed at different time points after PBMC transduction by stimulating T-body-hPSMA/fluc with PC3-PIP hPSMA^+^ or PC3 hPSMA^−^ cancer cell lines. T-bodies unstimulated or treated with PMA/Ionomycin represented the negative and positive controls, respectively. Similar results were obtained with T-body-hPSMA/eGFP. (C) c-myc expression in T-body-hPSMA/fluc populations tested for IFN-γ production.

CAR expression provided the transduced population with the ability to recognize the hPSMA antigen on the surface of prostate tumor cell lines, and to mediate a high and specific cytotoxicity (Fig. 3A). Indeed, both the T-body-hPSMA/fluc and T-body-hPSMA/eGFP populations lysed the hPSMA-transfected PC3 cells while sparing the antigen-negative counterpart; more importantly, they were also capable of recognizing the LNCaP target cells that naturally harbour the hPSMA antigen. Cytotoxicity was already evident, albeit at low levels, 3 days after transduction and increased at maximal levels just after a single round of antigen restimulation, to remain constant thereafter up to 2 months post-infection. Other than exerting a relevant cytotoxic activity, CAR-transduced T cells at different stages of differentiation also produced high levels of IFN-γ in response to hPSMA-expressing tumor cells (Fig. 3B and 3C), but not against hPSMA negative control cells. As a negative control, uninfected T cells did not produce IFN-γ when co-cultured with PC3 cells engineered to express the surface hPSMA antigen.

### Analysis of therapeutic efficacy of anti-hPSMA T-body administration against local prostate tumors

**Figure 4:**
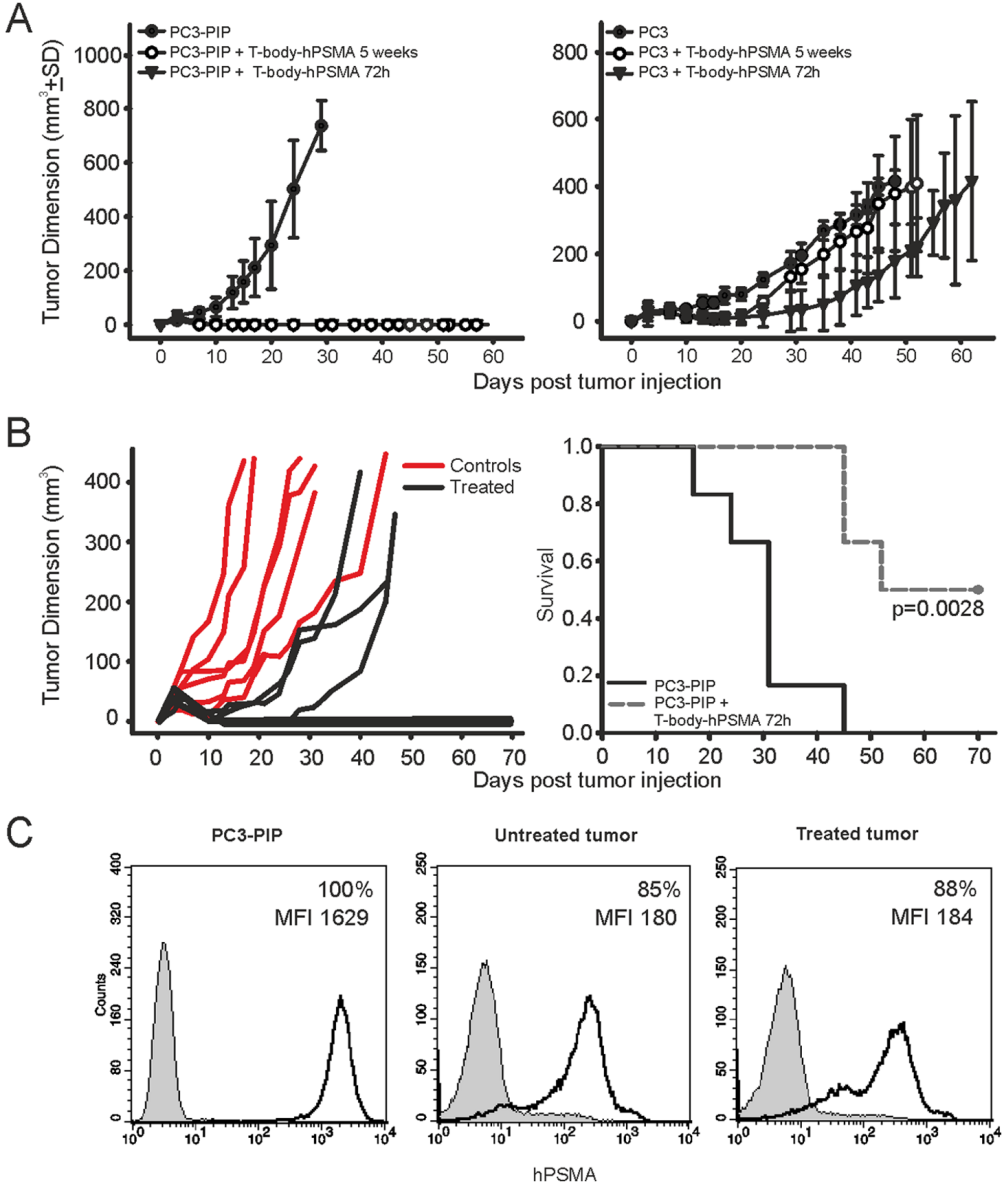
Assessment of T-body *in vivo* loco-regional therapeutic efficacy. (A) Winn Assay. PC3-PIP (*left panel*) and PC3 (*right panel*) tumor cells were inoculated s.c. in SCID mice, alone or mixed 1∶1 with T-body-hPSMA/eGFP at opposite flanks of the same animal. Tumor growth was monitored over time by caliper measurement. Number of mice per group, n = 6. (B) Loco-regional therapy. T-body-hPSMA/eGFP at 72 hours post transduction were administered intralesionally and perilesionally in SCID mice 4 days after s.c. injection of PC3-PIP tumor cells (n = 6); untreated animals served as control group (n = 6). *Left panel* shows tumor volumes, while *right panel* reports Kaplan-Meyer survival curves of treated and untreated mice. (C) Expression of hPSMA antigen in prostate tumors. PC3-PIP tumor cells from *in vitro* cultures (first quadrant) or isolated *ex-vivo* from control or treated mice (second and third quadrant, respectively; dark line) were evaluated for hPSMA expression by flow cytometry. The grey plot corresponds to the isotype control.

*In vivo* therapeutic efficacy of CAR-transduced T cells at different stages of differentiation (72 hours or 5 weeks post-transduction) was initially evaluated using a Winn assay (Fig. 4A). In particular, when PC3-PIP tumor cells were co-injected with T-body-hPSMA/eGFP no tumor growth was observed (Fig. 4A, *left panel*), irrespective of the differentiation stage of T-bodies. On the other hand, T cell transfer did not significantly impact the growth of hPSMA-negative PC3 tumor cells (Fig. 4A, *right panel*).

Since the Winn assay does not recapitulate a real therapeutic setting, we assessed the antineoplastic activity of CAR-transduced T cells against established tumors. SCID mice were injected s.c. with PC3-PIP cells and, when tumors became palpable, they received intratumorally and peritumorally T-body-hPSMA/eGFP at 72 hours post-transduction. While progressive tumor growth was observed in untreated control mice, treated masses shrank up to almost disappear, at least for the first week after treatment (Fig. 4B, *left panel*). Thereafter, tumors resumed to grow in 3 out of 6 treated mice, even though with a strong delay and a slower kinetics (Fig. 4B, *left panel*). Nevertheless, a single injection of CAR-transduced T cells produced a complete regression of the tumor in 50% of treated animals (3/6) and a statistically significant increase in survival (p = 0.0028; Fig. 4B, *right panel*).

To assess if the resumption of tumor growth could be due to the emergence of antigen-loss escape variants induced by the selective pressure of the immunological treatment, we analyzed the expression of hPSMA on tumor cells recovered *ex vivo* from treated and untreated mice (Fig. 4C). Indeed, this hypothesis could be ruled out because *ex vivo* cancer cells from both treated and control mice displayed overlapping profiles of hPSMA expression. Nonetheless, these expression levels turned out to be strongly reduced respect to the cultured tumor cells, as evidenced by a one-log decrease in the fluorescence intensity signal.

Finally, T-body-hPSMA/fluc were administered systemically to mice bearing s.c. tumors, but did not display therapeutic activity (Fig. S1A). This failure was very likely due to the poor capacity of infused T cells to reach the tumor site (Fig. S1B). Such result is in line with previous data obtained by our group in a different experimental setting, which involved the i.v. administration of Melan-A/MART-1-specific TCR-transduced T cells in mice bearing established s.c. melanoma tumors [20].

### Assessment of therapeutic efficacy of anti-hPSMA T-body administration against disseminated prostate tumors

While the loco-regional approach provided evidence of efficacy and specificity, a systemic treatment against a disseminated prostate tumor model would better resemble the clinical setting. To this end, different numbers of PC3-PIP cells were injected i.v. in SCID mice and tracked for their persistence and biodistribution by BLI; unexpectedly, they apparently disappeared very rapidly and failed to form detectable tumor masses up to one month after injection (Fig. S2 and data not shown). Therefore, tumor growth was then tested in two more immunocompromised animal strains, namely Rag2^−/−^/γc^−/−^ and NOD/SCID mice. In such strains, tumor cells disseminated and displayed a sustained growth as soon as 7–14 days after injection, even when administered at low amounts (Fig. S2).

**Figure 5:**
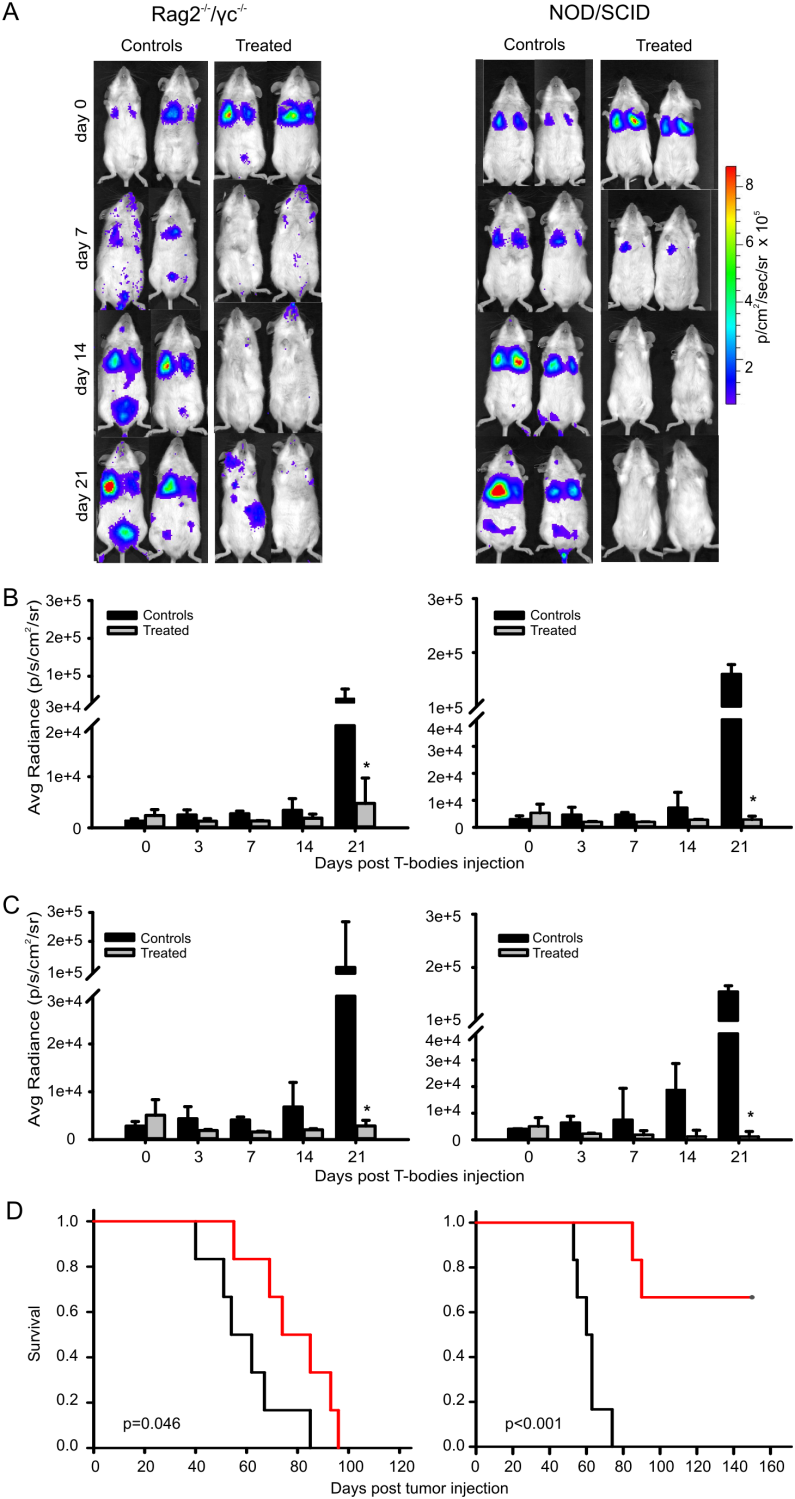
Assessment of T-body *in vivo* systemic therapeutic efficacy against disseminated prostate carcinoma. Rag2^−/−^/γc^−/−^ mice (n = 6) and NOD/SCID mice (n = 6) bearing established bioluminescent PC3-PIP tumors were injected i.v. with 2×10^7^ T-body-hPSMA/eGFP for 3 times at a two day interval. Untreated animals (n = 6 for both mouse strains) were used as controls. (A) Pictures show two representative Rag2^−/−^/γc^−/−^ and NOD/SCID mice (*left* and *right panels*, respectively) imaged by BLI at different time points, whereas (B) and (C) graphs report cumulative results of the regions of interest (ROI) in lungs and in total body, respectively. Tumor growth was monitored as photon flux and quantified as photon * sec^−1^ * cm^−2^ * sr^−1^. Graphs show mean ± SD of three independent experiments. *: P<0.05. The t-Test was used for statistical analysis. (D) Cumulative Kaplan-Meier survival curves of Rag2^−/−^/γc^−/−^ (*left panel*; untreated mice, black line; median survival = 54 days; treated mice, red line; median survival = 74 days; P = 0.046) and NOD/SCID mice (*right panel*; untreated mice, black line; median survival = 60 days; treated mice, red line; median survival = not evaluable; P<0.001).

As a complimentary approach, biodistribution and persistence of i.v.-administered T-body-hPSMA/fluc was also tested in healthy individuals of these mouse strains. In SCID mice, T cells disappeared just few hours after injection; conversely, T cell survival appeared slightly increased in Rag2^−/−^/γc^−/−^ mice, although the BLI signal was detectable only in the lungs; on the other hand, bioluminescence in NOD/SCID mice distributed uniformly throughout the body and could be tracked up to seventy-two hours (Fig. S3 and data not shown). Based on this evidence, we assessed the systemic therapeutic efficacy of T-bodies in Rag2^−/−^/γc^−/−^ and NOD/SCID mice (Fig. 5). Animals were injected i.v. with bioluminescent PC3-PIP cells and treated with 2×10^7^ T-body-hPSMA/eGFP for 3 times at 2 day intervals. In both mouse strains, this ACT approach strikingly reduced the BLI signal in lungs of treated animals as compared to control mice (Fig. 5A and 5B). Notably, while in Rag2^−/−^/γc^−/−^ mice bioluminescent lesions involving extra-pulmonary sites ultimately appeared, no metastatic disease become apparently evident in NOD/SCID mice (Fig. 5A and 5C), likely due to the total body diffusion of transferred T-bodies (Fig. S3). These results reflected on the long-term survival of treated mice (Fig. 5D). Indeed, the transfer of CAR-transduced T cells significantly improved survival in both strains of mice; more importantly, the treatment afforded to completely eradicate the neoplasia in more than 60% of NOD/SCID animals (4/6), which were completely disease-free 150 days after tumor induction.

## Discussion

The high curative potential against hematologic malignancies afforded by CAR T cells support the large number of clinical trials targeting these entities (36 out of 62 trials retrieved at www.clinicaltrials.gov by using the following key words: “Chimeric Antigen Receptor”, “CAR”, “T-Body” and “Designer T cells”), and in particular those expressing the CD19 antigen (29/36). Conversely, the difficulties intrinsic to the treatment of solid tumors (in particular the choice of suitable and safe target antigens) and the largely unsatisfactory results obtained thus far make them a less appealing but still open field of battle for immunotherapeutic strategies.

Here, we propose a second generation anti-hPSMA CAR based on a novel and high affinity scFv [19], and provide evidence of a strong and specific antitumor activity by the resulting T-bodies-hPSMA against a prostate tumor model, not only *in vitro* but also *in vivo*; in particular, we completely eradicated a disseminated neoplasia in more than 60% of treated NOD/SCID animals. This experimental design appears to recapitulate more realistically a clinical situation than the systemic treatment of localized tumors (s.c. tumors or pulmonary metastatic models [13], [16], [17], [26]); indeed, while the efficacy of our approach against localized tumors may appear reduced in comparison to other reported models [13], [16], [26], results of our systemic treatment against disseminated neoplasia reproduce or even outperform previously published data. As an example, the infusion of 3^rd^ generation anti-PSCA CAR-transduced T cells induced only a tumor growth delay, albeit statistically significant [16].

Interestingly, the therapeutic superiority of 3^rd^ over 2^nd^ generation CAR is far from being undoubtedly demonstrated. Conflicting evidences came from pre-clinical studies [17], [27], and only a pilot clinical trial involving 3^rd^ generation CAR against CD20^+^ B-cell malignancies has been published thus far [28]. In fact, no definitive conclusions can be drawn because of the low number of enrolled patients and the very low CAR expression obtained through plasmid electroporation (below the limit of detection by flow cytometry and Western blotting), which precludes the realistic assessment of potential adverse events. This safety aspect acquires even more importance in the case of the addition of the 4-1BB moiety, which can induce not only antigen-dependent but also antigen-independent proliferation [29], thus carrying additional risks of cell-mediated acute toxicity. Moreover, an excess of stimulation can be detrimental to modified cells, due to the potential induction of activation-induced cell death (AICD), as demonstrated for 3^rd^ generation CAR-transduced CIK cells [30].

On the other hand, 3^rd^ generation CAR appeared to prolong T cell survival at least in preclinical models [17], [26]. In a clinical setting, this issue will be addressed by the side-by-side comparison between 3^rd^ and 2^nd^ generation CAR-T cells in a currently recruiting clinical trial (SAGAN, NCT01853631), as previously assessed for the 2^nd^ and 1^st^ generation CAR-T cells [31]. Nonetheless, in our hands a second generation CAR design appeared to deliver very balanced activation/costimulatory signals, which were sufficient to sustain the *in vivo* persistence of T cells at detectable levels until the therapeutic activity became evident. This result was achieved despite the fact that our T-bodies displayed a sub-optimal effector phenotype at the time of transfer and did not receive any additional help. Indeed, they operated without exogenous IL-2 supply and in the virtual absence of CD4^+^ T cells (<5%). In this regard, it is noteworthy that the percentage of CD4^+^ T cells appeared to positively correlate with the clinical outcome in neuroblastoma patients treated with GD2-CAR specific T cells [8], and other protocols comprise the addition of unmodified CD4^+^ T cells to the infusates [27].

Moreover, the elevated and sustained expression of high affinity CAR molecules likely concurred to the outcome, leading to compensate for the reduced PSMA expression on target cells *in vivo*. A down-modulation of Ag expression is not an unexpected circumstance for human cell lines upon *in vivo* transfer in a mouse host [32], [33]. Accordingly, a reduced Ag expression can potentially jeopardize the final outcome, since the intensity of Ag expression has been correlated with the kinetic of killing performed by CAR-T cells, and ultimately with their capacity to completely eradicate a tumor [34].

The transgene expression level was achieved by means of a lentiviral vector with a very efficient bidirectional promoter [23]. This same lentiviral vector, albeit carrying a different CAR molecule, performed equally well compared to a related retroviral construct [35] in terms of transgene expression levels and antitumor activity. In addition, a lentiviral vector was chosen instead of the more widely used retroviral constructs because of technical considerations (less significant gene expression silencing due to position effects [29]), and the safety profile. Indeed, the use of retroviral vectors can be afflicted by a considerable risk of leukemogenesis, as recently demonstrated in a clinical trial for Wiscott-Aldrich Syndrome [36]. The follow-up for clinical use of lentiviral vectors is at present too short to completely exclude genotoxicity, but data from experimental models, which were predictive for retroviral vectors, come out in favor of a less pronounced risk of insertional mutagenesis [37], [38].

Thanks to the bidirectional promoter, the lentiviral vector used allowed the simultaneous expression of the CAR and a reporter gene, and hence the visualization of T cells after transfer; thus, we could positively correlate the persistence and the total body diffusion of T cells with their therapeutic efficacy. Indeed, whereas transferred T cells were detected only in the lungs of Rag2^−/−^/γc^−/−^ mice, they disseminated uniformly throughout the body and persisted longer in the NOD/SCID strain. Accordingly, treated Rag2^−/−^/γc^−/−^ mice developed tumor masses in extra-pulmonary sites and ultimately died, while more than 60% of NOD/SCID mice were still alive 150 days after tumor induction and showed no metastatic disease at sacrifice.

Two main questions remain that cannot be adequately assessed *per se* by a xenogeneic model, i.e. the validation of the chosen target and the related risk of “on-target, off-site” toxicity [39]. Indeed, preclinical models did not predict the dramatic events that occurred after translation in clinical trials [40], [41]. In this regard, PSMA is physiologically expressed in kidney, nervous system glia, and small intestine [18], and the risk to damage these organs and structures cannot be excluded simply based on the fine target specificity demonstrated in an experimental setting by the novel mAb we used to design our CAR [19]. However, in a clinical scenario, this vector containing a bidirectional promoter could be exploited to address this safety issue, namely by driving the simultaneous expression of a CAR molecule and a “safety switch” suicide gene, such as an inducible caspase or the herpes simplex virus thymidine kinase (HSV-TK) [35], [42]; or alternatively a chimeric costimulatory receptor (CCR) for combinatorial antigen recognition [43].

In conclusion, the novel CAR developed can be envisaged as a potential new weapon in the arsenal against prostate tumor and reported data strongly support its clinical exploitation based on the potent *in vivo* anticancer activity.

## Supporting Information

Figure S1 **Assessment of therapeutic efficacy of i.v.-administered T-bodies against subcutaneous prostate tumors.** (A) T-body-hPSMA/fluc at 72 hours or 5–6 weeks post transduction were administered i.v. in SCID mice 4 days after s.c. injection of PC3-PIP tumor cells (n = 6); untreated animals served as control group (n = 6). (B) T-bodies-hPSMA/fluc were inoculated i.v. in SCID mice 4 days after s.c. injection of PC3-PIP (right flank) or PC3 (left flank) tumor cells; cell distribution was assessed at different time points thereafter. Two representative mice out of six are depicted.
(TIF)

Figure S2 **Set up of a disseminated prostatic tumor model.** Comparison of tumor growth in SCID (upper panels), Rag2^−/−^/γc^−/−^ (central panels) and NOD/SCID (lower panels) mouse strains. Different numbers of bioluminescent PC3-PIP cells were injected i.v., and their survival and distribution were assessed at different time points. Images of two representative mice for each group are shown.
(TIF)

Figure S3 **T-bodies biodistribution in healthy mice of different strains.** T-bodies-hPSMA/fluc (20×10^6^/mouse) were inoculated i.v. in SCID (left panels), Rag2^−/−^/γc^−/−^ (central panels) and NOD/SCID (right panels) mice; cell distribution and survival was assessed at different time points thereafter. A representative mouse for each group is depicted.
(TIF)
