# Supplementary figures and images for: PSMA-Specific CAR-Engineered T Cells Eradicate Disseminated Prostate Cancer in Preclinical Models
Source: PLoS One. 2014 Oct 3;9(10):e109427. doi: 10.1371/journal.pone.0109427 (PMC4184866; doi:10.1371/journal.pone.0109427)

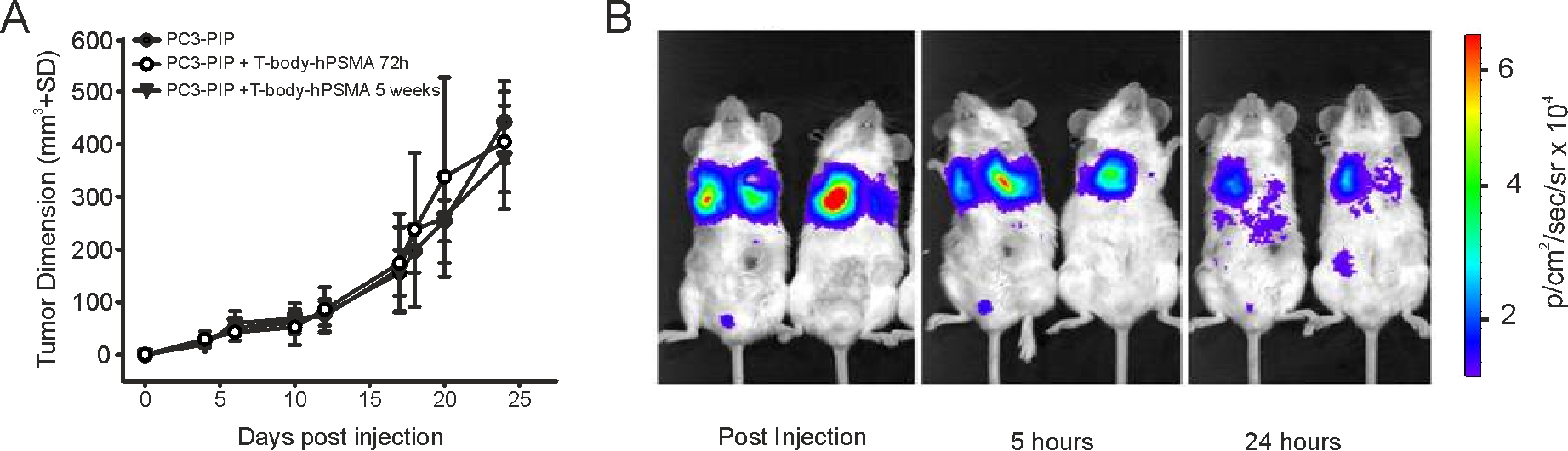

Supplement: Figure S1 — Assessment of therapeutic efficacy of i.v.-administered T-bodies against subcutaneous prostate tumors. (A) T-body-hPSMA/fluc at 72 hours or 5–6 weeks post transduction were administered i.v. in SCID mice 4 days after s.c. injection of PC3-PIP tumor cells (n = 6); untreated animals served as control group (n = 6). (B) T-bodies-hPSMA/fluc were inoculated i.v. in SCID mice 4 days after s.c. injection of PC3-PIP (right flank) or PC3 (left flank) tumor cells; cell distribution was assessed at different time points thereafter. Two representative mice out of six are depicted. (TIF) [file pone.0109427.s001.tif]

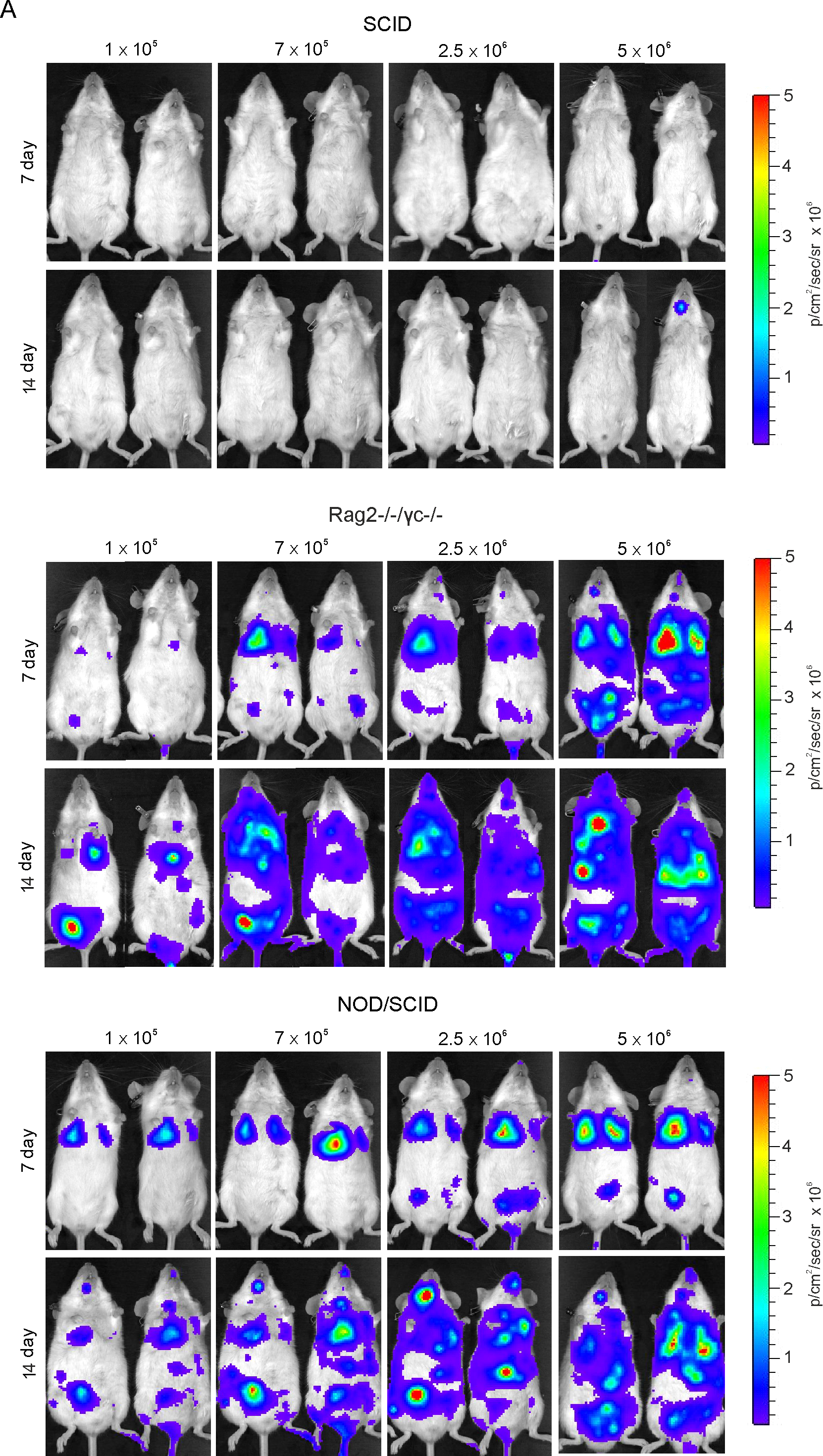

Supplement: Figure S2 — Set up of a disseminated prostatic tumor model. Comparison of tumor growth in SCID (upper panels), Rag2−/−/γc−/− (central panels) and NOD/SCID (lower panels) mouse strains. Different numbers of bioluminescent PC3-PIP cells were injected i.v., and their survival and distribution were assessed at different time points. Images of two representative mice for each group are shown. (TIF) [file pone.0109427.s002.tif]

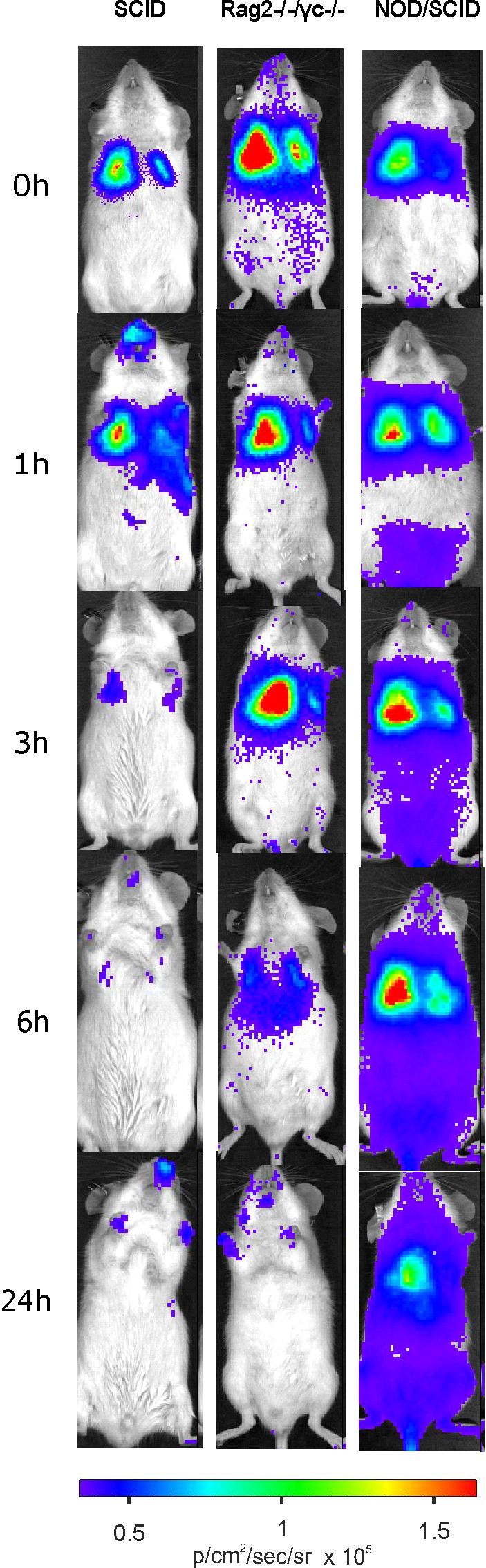

Supplement: Figure S3 — T-bodies biodistribution in healthy mice of different strains. T-bodies-hPSMA/fluc (20×106/mouse) were inoculated i.v. in SCID (left panels), Rag2−/−/γc−/− (central panels) and NOD/SCID (right panels) mice; cell distribution and survival was assessed at different time points thereafter. A representative mouse for each group is depicted. (TIF) [file pone.0109427.s003.tif]
